# Frequency of *TNFR1 36 A/G* gene polymorphism in azoospermic infertile men: A case-control study

**Published:** 2017-08

**Authors:** Hamid Reza Ashrafzadeh, Tahere Nazari, Masoud Dehghan Tezerjani, Maryam Khademi Bami, Saeed Ghasemi-Esmailabad, Nasrin Ghasemi

**Affiliations:** 1 *Medical Biotechnology Research Center, Ashkezar Branch, Islamic Azad University, Ashkezar, Yazd, Iran.*; 2 *Department of Medical Genetics, Tehran University of Medical Sciences, Tehran, Iran.*; 3 *Research and Clinical Center for Infertility, Yazd Reproductive Sciences Institute, Shahid Sadoughi University of Medical Sciences, Yazd, Iran. *; 4 *Recurrent Abortion Research Center, Yazd Reproductive Sciences Institute, Shahid Sadoughi University of Medical Sciences, Yazd, Iran.*

**Keywords:** Polymorphism, Male infertility, Spermatozoa, Cytokines, Tumor necrosis factor alpha

## Abstract

**Background:**

Tumor necrosis factor-alpha (*TNF*-α) is a multifunctional cytokine that regulates different cellular activities related to spermatogenesis. Tumor necrosis factor-alpha receptor 1 (*TNFR1*) mediates *TNF*-α activity and polymorphism in *TNFR1* could lead to gene dysfunction and male infertility.

**Objective:**

The aim of this study is to determine the association of *TNFR1* 36 A/G polymorphism with the idiopathic azoospermia in Iranian population.

**Materials and Methods:**

This case-control study included 108 azoospermic and 119 fertile men. This research investigated the frequency of *TNFR1* 36 A/G polymorphism in cases who were idiopathic azoospermic men referred to Yazd Research and Clinical Center for Infertility, Iran in comparison with controls. polymerase chain reaction- restriction fragment length polymorphism (PCR-RFLP) method was used to investigate the polymorphism in both case and control groups. PCR fragments were digested by Mspa1I enzyme and products were appeared by gel electrophoresis. The abundance of A→G was calculated in the azoospermic and healthy men.

**Results:**

According to the present study, GG and AG genotypes frequency in the azoospermic men group were higher than the control group (OR= 2.298 (1.248-4.229), p=0.007), (OR=1.47 (0.869-2.498, p=0.149). Our findings also showed that G allele frequency in azoospermic men had significant difference compared to the control group (OR=2.302 (1.580-3.355), p<0.001).

**Conclusion:**

It seems that the GG genotype and G allele have an association with increased risk of non-obstructive azoospermia.

## Introduction

Infertility is defined as an inability of a couple to conceive after one year of unprotected sexual intercourse (1, 2). Several changes in sperm parameters could be seen in infertile men, such as reduced sperm count, low sperm motility and morphological abnormalities (3, 4). Genetic causes of infertility include chromosomal and gene disorders (5). 

Studies on specific genes in humans and experimental models have shown the impact of genetic factors on infertility (6). Tumor necrosis factor (TNF) family is a group of cytokines that causes cell apoptosis. The most famous member of this family is tumor necrosis factor- alpha (TNF-α) that is derived from monocytes. The TNF-α is a multifunctional cytokine that regulates different cellular activities related to spermatogenesis. The TNF-α has two types of receptors: TNFR1 and TNFR2. These receptors have 2-6-repeated cysteine-rich motifs (7). The TNFR1 is characterized by its ability to induce cell death via an amino acid motif in the cytoplasmic domain called death domain (8). The TNFR1 contains cytoplasmic death domain that activates caspase cascade resulting in cell death (9). The TNFR1 is expressed in most tissues and it seems to be a key mediator in TNF signaling pathways (10, 11). The TNF-α is produced in testicular germ cells, especially in spherical spermatids, pachytene spermatocytes and testicular macrophages, as well as its receptors, are located on the Sertoli and Leydig cells (12-14). 

*TNFR1* gene is located on chromosome 12p13.2 (15). There are a large number of polymorphisms in this gene that can have a huge impact on the performance of the receptor; one of these polymorphisms is 36 A/G. Since this receptor plays a major role in the TNF-α activity, so it is important to investigate the polymorphisms associated with this gene. This research examined the association of alleles and genotypes of TNFR1 36 A/G polymorphism with sperm abnormalities. 

The aim of this study was to evaluate this polymorphism in Iranian male population with azoospermic disorders referred to the Recurrent Abortion Clinic of Yazd Reproductive Sciences Institute.

## Materials and methods

This analytical case-control study was conducted on 227 men referred to the Recurrent Abortion Clinic of Yazd Reproductive Sciences Institute Yazd, Iran between February 2013 to June 2013. A total of 108 non-obstructive azoospermic infertile men (age range 20-50 yr) were enrolled as the case group and 119 normal fertile men as the control group. 

Cases diagnosed by urologists after semen analysis and clinical examination in Research and Clinical Center for Infertility according to criteria of World Health Organization (19). Cases had normal karyotypes and normal hormonal assay. Control groups were fertile men with at least one normal child, who were chosen after routine check up test.

**Sampling**

Five ml of peripheral blood sample was collected in tubes containing anticoagulant EDTA (ethylenediaminetetraacetic acid) from each participant. Salting out method was used for DNA extraction. Extracted DNA quality was examined using Nanodrop device and the samples within the standard range were selected. The target gene was amplified using specific primers and polymerase chain reaction (PCR) techniques. PCR products were transferred onto agarose gel after affecting the restriction enzyme. The results of electrophoresis were analyzed. Table I and II show the optimized processes of PCR reaction. PCR reaction was performed to amplify the *TNFR1* gene fragment, and then electrophoresis was done on 2% agarose gel that 183-bp band was observed. The enzymatic digestion was carried out by the MSPA1I enzyme, then the samples were electrophoresed on 3% agarose gel.

**Digestion by MSPA1I enzyme**

After PCR reactions, the final analysis was performed by PCR-RFLP method with the help of restriction MSPA1I enzyme for all participants. After optimizing the enzymatic digestion conditions, samples were cut and then were taken on 3% agarose gel by enzyme MSPA1I (New England Biolabs (Neb) Co. Catalog no. R0577S). The length of amplified products containing this polymorphism was 183 base pairs. In the present study, three different patterns were expected to be observed on gel electrophoresis, including homozygous individuals (AA): a band in 183-bp region, heterozygous individuals (AG): three bands in 183-bp, 108-bp and 75-bp regions, as well as homozygous individuals (GG): two bands in 108-bp and 75-bp regions. 

**Ethical committee**

Ethical committee in Yazd Reproductive Sciences Institute approved the methods of this study and informed consent form was signed by all participants before starting this study.

**Statistical analysis**

After obtaining the relative frequency of alleles and genotypes in both studied groups (infertile and control), significant levels of frequencies, the difference between the two groups and their relationships with azoospermia were assessed by chi-square test using Statistical Package for the Social Sciences, version 20, SPSS Inc, Chicago, Illinois, USA (SPSS). P-value, Odds Ratio and OR Confidence Interval were calculated for the frequencies and parameters. 

## Results

In the present study, 108 non-obstructive azoospermic men and 119 controls were participated. The Mean age of cases and controls were 33.67 and 33.25 years respectively (p=0.104). Minimum and maximum duration of infertility in patients were between 2 and 20 years.

Three different patterns were observed for bands appeared on gel electrophoresis, including AA genotype (183 bp), AG genotype (183bp, 108bp, and 75bp) and GG genotype (108bp and 75bp). Figure I depict the image of digested products on 3% agarose gel. The results indicated that the frequencies of AA, AG and GG genotypes were 17.6%, 48.2%, and 34.3% respectively in cases and 42.8%, 38.7% and 18.5% in controls. In addition, the frequencies of A and G alleles were 41.7% and 58.3% respectively in case, and 52.1% and 47.9% in control group (Table IV). 

Present results demonstrated that the frequency of GG genotype in cases and controls was significantly different (p=0.007).The frequency of GG genotype was higher in cases than controls. The frequency of A and G alleles of TNF R1 36 A/G polymorphism was significantly different between cases and controls, and the risk of non-obstructive azoospermia was greater in men with GG genotype (OR=2.298, CI 1.24-4.229). Also, the difference in A and G alleles frequency was significant between groups (p<0.001) and the risk of azoospermia in people with G allele was higher (OR=2.302, CI 1.58-3.355) (Table IV).

**Table I.** Concentration and volume of the solutions in PCR reaction

**No.**	**Volume used in 25 µl**	**Substances**	
1	12.5 µl	2X Taq DNA Polymerase Master Mix RED-1.5mM MgCl_2_	
2	2	*TNFR1*F: GAGCCCAAATGGGGGAGTGAGAGG	Forward Primer
		*TNFR1*R: ACCAGGCCCGGGCAGGAGAG	Reveres Primer
3	1 µl	Genomic DNA	
4	9.5 µl	H_2_O	
5	Total volume= 25 µl		

**Table II:** PCR thermal profile for amplification of single nucleotide 36 A/G polymorphisms of *TNFR1* gene

**Temperature (** **ºC** **)**	**Time (minute)**	**Number of cycles**	**PCR ** **steps**
95	10	1	Initial denaturation
95 64 72	1 1 1	35	Denaturation Primer matching Elongation
72	5	1	Final elongation

PCR: Polymerase chain reaction

**Table III:** Frequency distribution of *TNFR1* 36 A/G polymorphism genotypes in infertile men with azoospermia (AA genotype was considered as reference

**Genotypes**	**Azoospermic men (n=108)**	**Controls ** **(n=119)**	**p-value**	**OR (CI; 95%)**
AA	19 (17.6)	51 (42.8)		
AG	52 (48.2)	22 (38.7)	0.149	1.47 (0.869-2.498)
GG	37 (34.3)	46 (18.5)	0.007	2.298 (1.248-4.229)

Chi-square test showed significant differences between genotypes frequencies in cases and controls

OR=2.298, CI; 95%, 1.248-4.229.

**Table IV:** Frequency distribution of *TNFR1* 36 A/G polymorphism alleles in patients with azoospermia (A allele was considered as reference)

**Genotypes**	**Azoospermic men (n=216) n (%)**	**Controls ** **(n=238)** **n (%)**	**p-value**	**OR (CI; 95%)**
A	90 (41.7)	124 (52.1)		
G	126 (58.3)	114 (47.9)	< 0.001	2.302 (1.58-3.355)

Chi-square test showed significant differences between alleles frequency in cases and controls

OR< 0.001, CI; 95%, 1.58-3.355

**Figure 1:**
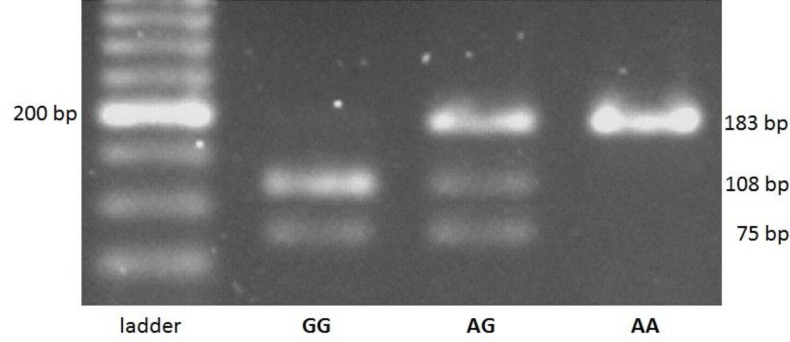
Image of products digested by a MSPA1I enzyme on an agarose gel.

## Discussion

This study was conducted to assess this polymorphism and its prevalence among patients with idiopathic azoospermia. Results of the present study showed G allele and GG genotype of TNFR1 36 A/G polymorphism is significantly higher in azoospermic infertile men. There are numerous reasons for infertility, which may be related to abnormalities in male or female or both (20). The infertility etiology remains unknown in 30 percent of infertile men (1). The environmental and genetic factors play a role in infertility, which nowadays the role of genetic factors has been confirmed in men with idiopathic infertility (21). 

It is estimated that about 40-50% of men have qualitative and quantitative abnormalities in their sperms (2). Spermatogenesis is depend on a large number of cellular signals causing coordination and connection between different cells of the testes. Cytokines, including TNF-α, regulate this relationship and function among the cells and, therefore it could be associated with sperm abnormalities, such as changes in morphology, number, and motility. TNF-α receptors such as TNFR1 and TNFR2 exist in sertoli and leydig ells. The function of these cells can be adjusted by binding the cytokine to them (22, 23). Evaluation of the polymorphisms of genes involved in spermatogenesis is one of the research areas in the genetics of male infertility. These polymorphisms can affect spermatogenesis and male fertility, such as 36 A/G polymorphism of TNFR1. This polymorphism can change in function of the type 1 receptor of TNF-α cytokine, resulting in cytokine dysfunction. Hence, the study of different variants of genes encoding these cytokine receptors leading to incomplete, more or less production of these proteins can be useful for causes of infertility and its treatment. 

Hosono et al examined the expression levels of TNF-α and its receptors in patients with Colorectal Adenoma. They determined that serum levels of TNFR1 were significantly higher in these patients compared with the control group, as well as found no significant correlation between serum levels of TNF-α and TNFR2 between patients and control group (24). The serum TNFR1 level in patients with Colorectal Adenoma significantly elevated compared to the control group. The results indicate the involvement of G allele with enhanced expression of TNFR1 and thus increased the function of TNF-α cytokine, confirming the significant association between G allele and the GG and AG genotypes and the possibility of high risk of azoospermia.

Tronchan et al in France population examined the relationship between 36 A/G polymorphism of TNFR1 with male infertility and sperm abnormalities using PCR-RFLP and ASPCR methods. The results demonstrated that allele A of TNF-α is associated with sperm motility and count, which increases the TNF-α expression (22). TNF-α affects both sperm concentration and motility via different pathways negatively. The mechanisms are alterations in miosis and release of spermatozoa into the seminiferous lumen via reconstruction of blood- testis barrier as the same as ectoplasmic changes in the communication of sertoli cells and spermatids. 

In addition, TNF-α decreases germ cell apoptosis through suppressing Fas ligand system. On the other hand, increase of TNF-α level is correlated with sperm DNA chromatin abnormalities and reduction of testosterone synthesis. Increased expression of TNF-α is associated with impaired sperm motility and count. For this reason and due to the role of TNFR1 as the TNF-α receptors, it is expected to be observed the effects similar to the effect of promoted TNF-α by increasing expression of this receptor. Bialas and colleagues in 2009 investigated the role of IL-6, IL-10, TNF-α-, TNFR1, and TNFR2 in the processes of normal and abnormal spermatogenesis as well as testicular tumors using Real-Time PCR technique. 

Their study showed a significant relationship between promoted expression of IL-6 and testicular tumors, but no association about IL-10. In addition, it was observed that TNFR1 expression levels increased significantly in patients with impaired sperm maturation compared with patients with testicular tumors. However, no significant correlation was found in the TNFR2 (25). In comparison with this study, our results also determined that high TNFR1 expression levels were significantly correlated with the risk of azoospermia. Lazaros et al examined 36 A/G polymorphism of TNFR1 in oligospermic male population. Their study confirmed a significant relationship between the allele G frequency and elevated risk of men to oligospermia (9). 

## Conclusion

The findings of the present study revealed a significant increase in G allele among patients with azoospermia compared with normospermic male. It can be concluded that the elevation in G allele might be effective in increasing male azoospermia.

## Note

***This article extracted from M.Sc. thesis. (Hamid Reza Ashrafzadeh)***

## References

[1] Pashaiefar H, Sheikhha MH, Kalantar SM, Jahaninejad T, Zaimy MA, Ghasemi N (2013). Analysis of MLH3 C2531T polymorphism in Iranian women with unexplained infertility. Iran J Reprod Med.

[2] Zaimy MA, Kalantar SM, Sheikhha MH, Jahaninejad T, Pashaiefar H, Ghasemzadeh J (2013). The frequency of Yq microdeletion in azoospermic and oligospermic Iranian infertile men. Iran J Reprod Med.

[3] Sheikhha MH, Zaimy MA, Soleimanian S, Kalantar SM, Rasti A, Golzade M (2013). Multiplex PCR Screening of Y-chromosome microdeletions in azoospermic ICSI candidate men. Iran J Reprod Med.

[4] Iammarrone E, Balet R, Lower AM, Gillott C, Grudzinskas JG (2003). Male infertility. Best Pract Res Clin Obstet Gynaecol.

[5] Talebi A, Vahidi S, Aflatoonian A, Ghasemi N, Ghasemzadeh J, Firoozabadi R (2012). Cytochemical evaluation of sperm chromatin and DNA integrity in couples with unexplained recurrent spontaneous abortions. Andrologia.

[6] Miyamoto T, Minase G, Okabe K, Ueda H, Sengoku K (2015). Male infertility and its genetic causes. J Obstet Gynaecol Res.

[7] Idriss HT, Naismith JH (2000). TNF alpha and the TNF receptor superfamily: structure-function relationship(s). Microscop Res Technique.

[8] Lysiak JJ (2004). The role of tumor necrosis factor-alpha and interleukin-1 in the mammalian testis and their involvement in testicular torsion and autoimmune orchitis. Reprod Biol Endocrinol.

[9] Lazaros LA, Xita NV, Chatzikyriakidou AL, Kaponis AI, Grigoriadis NG, Hatzi EG (2012). Association of TNFalpha, TNFR1, and TNFR2 polymorphisms with sperm concentration and motility. J Androl.

[10] Haider S, Knofler M (2009). Human tumour necrosis factor: physiological and pathological roles in placenta and endometrium. Placenta.

[11] van Horssen R, Ten Hagen TL, Eggermont AM (2006). TNF-alpha in cancer treatment: molecular insights, antitumor effects, and clinical utility. Oncologist.

[12] Said TM, Agarwal A, Falcone T, Sharma RK, Bedaiwy MA, Li L (2005). Infliximab may reverse the toxic effects induced by tumor necrosis factor alpha in human spermatozoa: an in vitro model. Fertil Steril.

[13] Li N, Wang T, Han D (2012). Structural, cellular and molecular aspects of immune privilege in the testis. Frontiers Immunol.

[14] Theas MS, Rival C, Jarazo-Dietrich S, Jacobo P, Guazzone VA, Lustig L (2008). Tumour necrosis factor-alpha released by testicular macrophages induces apoptosis of germ cells in autoimmune orchitis. Hum Reprod.

[15] Hong CY, Park JH, Ahn RS, Im SY, Choi HS, Soh J (2004). Molecular mechanism of suppression of testicular steroidogenesis by proinflammatory cytokine tumor necrosis factor alpha. Mol Cell Biol.

[16] Agarwal A, Said TM (2003). Role of sperm chromatin abnormalities and DNA damage in male infertility. Hum Reprod Update.

[17] Khademi Bami M, Dehghan Tezerjani M, Montazeri F, Ashrafzadeh Mehrjardi HR, Ghasemi-Esmailabad S, Sheikhha MH (2017). Tumor Necrosis Factor Alpha -308 G/A Single Nucleotide Polymorphism and Risk of Sperm Abnormalities in Iranian Males. Int J Fertil Steril.

[18] Farivar Sh, Akhondi MM, Modarresi MH, Sadeghi MR (2009). GSTM1 and GSTP1 polymorphisms and glutathione S-transferase activity: Iranian infertile men. Tehran Univ Med J.

[19] Esteves SC (2014). Clinical relevance of routine semen analysis and controversies surrounding the 2010 World Health Organization criteria for semen examination. Int Braz J Urol.

[20] Farivar S, Dehghan Tezerjani M (2012). Human Leukocyte Antigen class Ib and pregnancy success. Int J Fertil Steril.

[21] Pasqualotto FF, Pasqualotto EB, Sobreiro BP, Hallak J, Medeiros F, Lucon AM (2006). Clinical diagnosis in men undergoing infertility investigation in a university hospital. Urologia Int.

[22] Tronchon V, Vialard F, El Sirkasi M, Dechaud H, Rollet J, Albert M (2008). Tumor necrosis factor-alpha -308 polymorphism in infertile men with altered sperm production or motility. Hum Reprod.

[23] Zalata A, Atwa A, El-Naser Badawy A, Aziz A, El-Baz R, Elhanbly S (2013). Tumor necrosis factor-alpha gene polymorphism relationship to seminal variables in infertile men. Urology.

[24] Hosono K, Yamada E, Endo H, Takahashi H, Inamori M, Hippo Y (2012). Increased tumor necrosis factor receptor 1 expression in human colorectal adenomas. World J Gastroenterol.

[25] Bialas M, Fiszer D, Rozwadowska N, Kosicki W, Jedrzejczak P, Kurpisz M (2009). The role of IL-6, IL-10, TNF-alpha and its receptors TNFR1 and TNFR2 in the local regulatory system of normal and impaired human spermatogenesis. Am J Reprod Immunol.

